# A Rare Case of Vascular Leiomyosarcoma Originating from a Branch Vessel of the External Iliac Vein

**DOI:** 10.1155/2018/5160591

**Published:** 2018-07-15

**Authors:** Akinaru Yamamoto, Wataru Nakata, Gaku Yamamichi, Go Tsujimura, Yuichi Tsujimoto, Mikio Nin, Hideaki Miwa, Masao Tsujihata

**Affiliations:** ^1^Department of Urology, Osaka Rosai Hospital, 1179-3 Nagasone, Kitaku, Sakai, Osaka 591-8025, Japan; ^2^Department of Pathology, Osaka Rosai Hospital, 1179-3 Nagasone, Kitaku, Sakai, Osaka 591-8025, Japan

## Abstract

Leiomyosarcoma arising from the external iliac vein is uncommon. This is a report of a 51-year-old Japanese man with venous leiomyosarcoma originating from a branch vessel of the left external iliac vein. The tumor was found during a medical examination, and the patient had no symptoms. Computed tomography showed a 72 × 49 mm mass adjacent to the left external iliac vein. The tumor was resected en-block along with ligation of the external iliac vein due to strong adhesion with the tumor. Histological examination showed venous leiomyosarcoma, and its origin was thought to be a branch vessel of the left external iliac vein. The patient has remained free from recurrence at 30 months after surgery.

## 1. Introduction

Leiomyosarcomas account for approximately 6% of all soft tissue sarcomas, and venous leiomyosarcomas account for 2% of all leiomyosarcomas [1]. Venous leiomyosarcomas commonly arise from the inferior vena cava, pulmonary vein, femoral vein, great saphenous vein, and jugular vein [2]. A venous leiomyosarcoma originating from a branch vessel of the left external iliac vein is rare, and this may be the first report of this finding. Generally, the prognosis of leiomyosarcoma is poor. Chemotherapy and/or radiation therapy can be performed to improve the prognosis, but their efficacy remains debatable. Only complete resection of the tumor is necessary for long-term survival. In the present case, there has been no evidence of recurrence at 30 months after complete surgical resection along with ligation of the left external iliac vein.

## 2. Case Presentation

**Figure 1:**
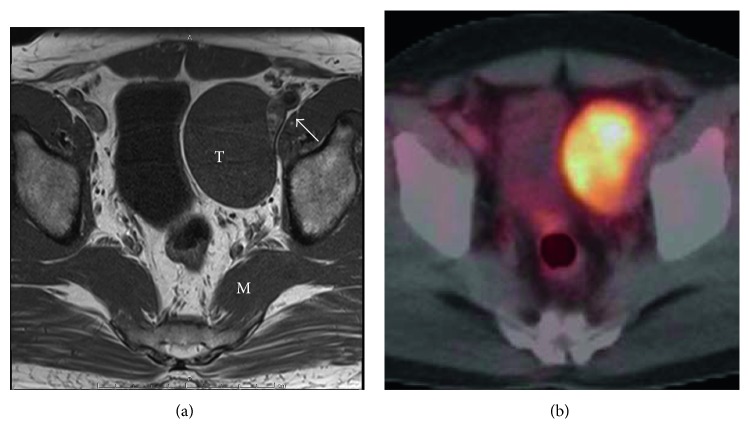
(a) Magnetic resonance imaging showed that the tumor (T) was isointense with muscle (M) in the T1-weighted image. The tumor adjoins the left external iliac vein (arrow). (b) Positron-emission computed tomography scan showed abnormal integration at the tumor site.

A 51-year-old Japanese man was referred to our hospital for abnormal ultrasound findings during a medical examination. He had no complaints or relevant family history. His past history included diabetes, hypertension, dyslipidemia, and fatty liver. Physical examination showed nothing of note. Laboratory findings, including serum concentrations of oncological markers such as alpha fetoprotein, cancer antigen 19–9, neuron-specific enolase, and carcinoembryonic antigen, were within the normal range. A contrast computed tomography (CT) scan revealed a 72 × 49 mm mass closely adjoining the left external iliac vein. Magnetic resonance imaging showed that the mass was isointense with muscle in the T1-weighted image (Figure 1(a)). A positron-emission computed tomography (PET-CT) scan showed abnormal integration at the tumor site (Figure 1(b)). These findings suggested that the tumor was a leiomyosarcoma probably derived from the left external iliac vein.

Before surgery, we discussed with the vascular surgeons how to deal with the left iliac vein. In general, blood vessel reconstruction and subsequent anticoagulation therapy are performed. However, the patient was engaged in physical labor and did not want to take an anticoagulant after surgery. So, we finally decided to perform combined resection of the left iliac vein with the tumor. Extirpation of the tumor was performed. We could easily peel the tumor from surrounding tissue except at the left external iliac vein where, as predicted, the tumor was strongly adhered. Therefore, complete tumor resection was achieved by combined resection of the external iliac vein.

**Figure 2:**
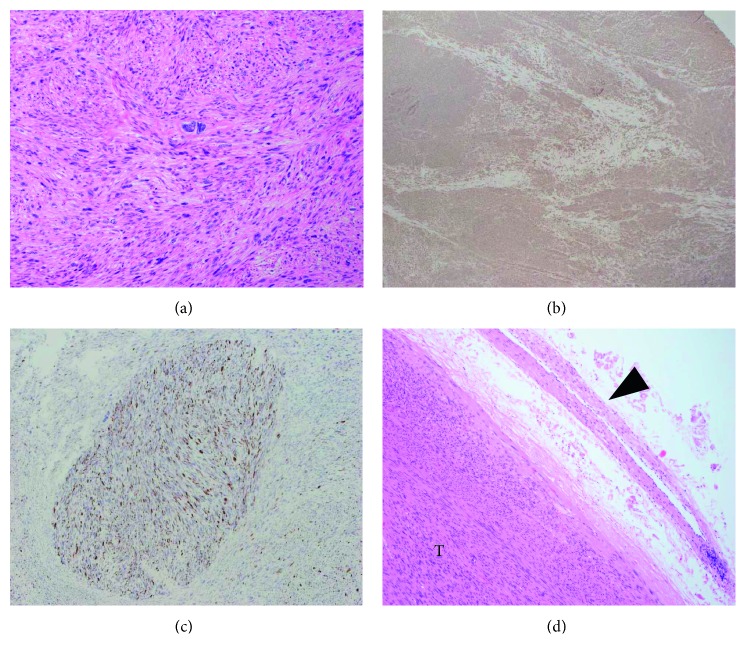
(a) There were areas with high cellularity and bizarre nuclei. (b) Immunostaining for *α*-SMA was positive. (c) The positive ratio of MIB-1 was low (5–10%). (d) The tumor (T) was separated from the external iliac vein (black arrowhead), and only fibrous adhesions without infiltration were present.

**Figure 3:**
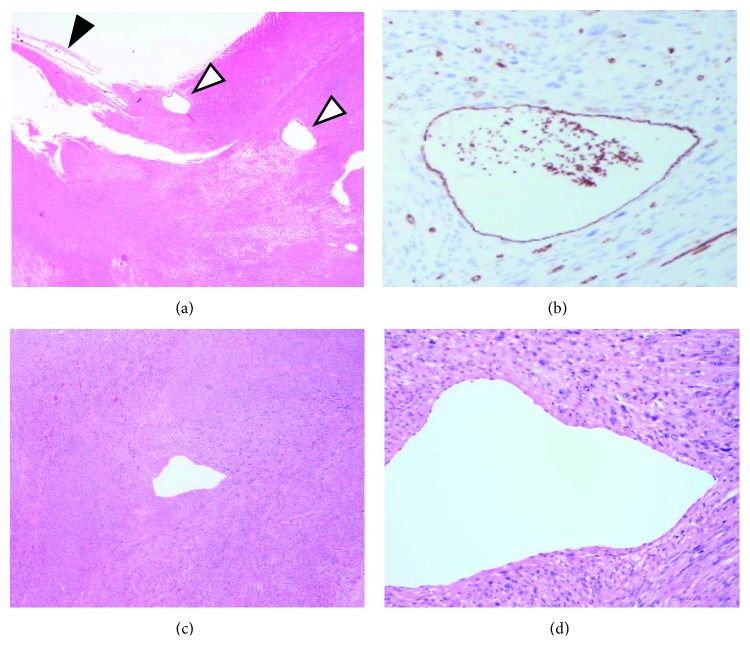
(a) There were thick blood vessels, which were thought to be branching vessels (white arrowheads) of the left external iliac vein (black arrowhead). (b) Immunostaining for CD31 was positive, and we could identify the vascular endothelium. (c) The tunica media and tunica externa of this vascular wall were diminished and completely replaced by tumor cells. (d) The vessel intima was completely replaced by tumor cells, and the vessel wall structure was diminished.

Pathological examination revealed a gross, well-defined, firm tumor of 60-mm at the greatest diameter. The cut surface was gray-white with a whorled appearance. Microscopically, the tumor was mostly composed of interlacing fascicles of spindle cells with a mild to moderate degree of cellular pleomorphism and was considered to be a low-grade leiomyosarcoma. Furthermore, there were focal areas of high cellularity and bizarre nuclei (Figure 2(a)). Immunostaining for *α*-SMA (Figure 2(b)) was positive, and that for S-100, c-kit, and DOG-1 were negative. The positive ratio of MIB-1 was low at 5–10% (Figure 2(c)). Contrary to expectation, the tumor was separated from external iliac vein, where only fibrous adhesions without infiltration were present (Figure 2(d)). There was a thick blood vessel in the tumor that was thought to be a branch vessel of the left external iliac vein (Figure 3(a)). Its vessel intima was preserved, and immunochemical staining for CD31 was positive (Figure 3(b)). However, the tunica media and tunica externa of this vascular wall were diminished and completely replaced by tumor cells (Figures 3(c) and 3(d)). Therefore, we definitively diagnosed venous leiomyosarcoma originating from a branch vessel of the left external iliac vein.

Immediately after surgery, the patient developed left leg pain and swelling. However, the painful swelling of his left thigh improved one week later and ultimately disappeared by about 3 months after surgery. The patient has remained free from recurrence at 30 months after surgery.

## 3. Discussion

Vascular leiomyosarcomas usually arise from a vein, approximately 5 times more often than from an artery [1, 3]. A preference for the female sex has been reported: 82.6% of all patients are female, and leiomyosarcomas usually appear around 50–60 years of age [4]. Primary venous leiomyosarcomas arise from vascular smooth muscle cells, and the growth pattern may progress from intramural to endoluminal, extraluminal, or mixed forms [5, 6]. The endoluminal pattern has a worse prognosis than the extraluminal pattern because of its high rate of metastasis.

Standard treatment for vascular leiomyosarcoma is surgical resection, and 40–60% of patients undergo surgery [7]. Optimal treatments with chemotherapy and radiotherapy have not yet been established. When curative surgical resection is performed, the rates of 3- and 5-year survival are 76% and 33%, respectively. If a curative operation is not performed, the prognosis is bad [8]. However, an aggressive surgical approach assuring a free en-bloc tumor resection can be curative despite a reported local recurrence rate of 53.7% in selected cases at an average of 25 months after surgery [4, 9, 10]. In our investigation, we found a few reports of leiomyosarcomas arising from the external iliac vein, but no report of leiomyosarcoma originating from a branch vessel of this vein. These reports showed that such leiomyosarcomas may have a relatively good prognosis. The reason for this may be that the tumor growth pattern likely progresses from intramural to extraluminal. When the tumor progresses endoluminally, symptoms such as leg pain, swelling, and leg numbness appear immediately due to constriction of the external iliac vein. For this reason, a leiomyosarcoma originating from the external iliac vein can be detected early and the patient can undergo a curative operation; thus, their prognosis is relatively good.

In most of the reports of vascular leiomyosarcomas originating from the external iliac vein, blood vessel reconstruction was performed. However, a recent report revealed that blood vessel reconstruction had a higher incidence of thrombosis while providing no additional benefit in reducing symptomatic extremity edema compared to ligation in patients with isolated external iliac vein injuries [11]. Furthermore, patients who underwent blood vessel reconstruction must permanently take anticoagulants. We ligated the left external iliac vein in our patient immediately after surgery; although he suffered left leg pain and swelling, it had resolved by 3 months later. He does not take anticoagulants. Thus, ligation of the external iliac vein as curative surgery for vascular leiomyosarcoma originating from a branch vessel of the external iliac vein may be one viable treatment option.
